# Actinium-225-rhPSMA-10.1 as a novel, alpha-particle-emitting therapy for prostate cancer: results of a preclinical evaluation

**DOI:** 10.3389/fonc.2026.1868487

**Published:** 2026-07-16

**Authors:** Caroline Foxton, Bradley Waldron, Alexander Wurzer, Calogero D’Alessandria, Alfred Morgenstern, Frank Bruchertseifer, Tea Kirkegaard Nielsen, Rikke Veggerby Grønlund, Mathias Wikke Hallund, Daniel J. Stevens

**Affiliations:** 1 Blue Earth Diagnostics Ltd, Oxford, United Kingdom; 2 Blue Earth Therapeutics Ltd, Oxford, United Kingdom; 3 Department of Nuclear Medicine, Technical University of Munich, Munich, Germany; 4 European Commission, Joint Research Centre, Karlsruhe, Germany; 5 Minerva Imaging, Ølstykke, Denmark

**Keywords:** actinium-225, lutetium-177, prostate cancer, PSMA-targeted therapy, radiohybrid pharmaceutical

## Abstract

**Background:**

Novel, radiohybrid, beta-particle-emitting, PSMA-targeted radiopharmaceutical therapy [^177^Lu]Lu-rhPSMA-10.1 shows promising preclinical and clinical efficacy in prostate cancer. Here, we evaluated [^225^Ac]Ac-rhPSMA-10.1 as a potential alpha-particle-emitting, PSMA-targeted radiopharmaceutical therapy for prostate cancer.

**Methods:**

Lipophilicity of [^225^Ac]Ac-rhPSMA-10.1 and [^177^Lu]Lu-rhPSMA-10.1 (0.1-0.3 MBq) was assessed by shake-flask method. PSMA-binding affinity (IC_50_) was evaluated in a competitive binding assay using rhPSMA-10.1 complexed with natural Lanthanum ([^nat^La]; non-radioactive [^225^Ac]Ac surrogate) or natural Lutetium ([^nat^Lu]) in PSMA-expressing LNCaP cells. Cellular internalization (normalized to that of [^177^Lu]Lu-PSMA-I&T) was assessed after 1-hour incubation of LNCaP cells (5x10^5^ cells in 1 mL/well) with [^225^Ac]Ac-rhPSMA-10.1 or [^177^Lu]Lu-rhPSMA-10.1. *In vivo* assessments were conducted using PSMA-expressing 22Rv1 prostate cancer xenografts (eight mice/group plus untreated controls). Efficacy (tumour growth and survival) versus controls was assessed ≤49 days after a single-dose of [^225^Ac]Ac-rhPSMA-10.1 (30 KBq) or [^177^Lu]Lu-rhPSMA-10.1 (30 MBq). Tolerability was evaluated by bodyweight monitoring.

**Results:**

[^225^Ac]Ac-rhPSMA-10.1 and [^177^Lu]Lu-rhPSMA-10.1 showed similar *in vitro* profiles: low lipophilicity (log D_oct/PBS pH 7.4_ = -3.4 ± 0.2 and log D_oct/PBS pH 7.4_ = -3.8 ± 0.1, respectively), high PSMA binding affinity ([^nat^La]La-rhPSMA-10.1 IC_50_ = 3.6 ± 0.6 nM, [^nat^Lu]Lu-rhPSMA-10.1 IC_50_ = 1.6 ± 0.1 nM) and high internalization (99% ± 14 and 108% ± 5, respectively). *In vivo*, [^225^Ac]Ac-rhPSMA-10.1 significantly reduced tumour growth versus controls from Day 14-31 (*p* < 0.05), and significantly prolonged median survival (43.5 days) versus controls (27.0 days; *p* = 0.006). There were no significant differences in tumour growth suppression or survival between the radiopharmaceuticals, and both were well tolerated.

**Conclusion:**

[^225^Ac]Ac-rhPSMA-10.1 displayed similar *in vivo* properties to [^177^Lu]Lu-rhPSMA-10.1 and similar efficacy in prostate cancer xenografts at lower administered radioactivity.

## 1 Introduction

Prostate cancer is the most common cancer in men in the United States, with 313,780 new cases estimated to occur in 2025 (1). Despite therapeutic advances to slow disease progression and prolong survival, metastatic prostate cancer ultimately remains incurable (2, 3), thereby necessitating the development of alternative treatments.

Prostate-specific membrane antigen (PSMA) is overexpressed in prostate cancer cells, which makes PSMA-targeted radiopharmaceutical therapy (RPT) an attractive option for metastatic castration-resistant prostate cancer (mCRPC) (4). In the United States, Lutetium-177 ([^177^Lu]Lu) vipivotide tetraxetan is approved for the treatment of men with PSMA-positive mCRPC who have been treated with an androgen receptor pathway inhibitor, before or after taxane-based chemotherapy (2, 5–7). [^177^Lu]Lu vipivotide tetraxetan has provided an important additional option for men with few treatment options; nevertheless, it is estimated that approximately one-third of patients do not respond, and a further one-third progress quickly after an initial response, to [^177^Lu]Lu-labelled PSMA-targeted RPT (4, 8).

The decay characteristics of radioisotopes may influence their suitability for treating the different features of prostate cancer (9). For instance, beta particles (such as [^177^Lu]Lu) have a longer range and lower linear energy transfer, and their path length encompasses a much larger number of cells than alpha-particle-emitting radiation (10, 11). Therefore, beta particles may be more suitable for managing target expression heterogeneity. By contrast, alpha particles (such as Actinium-225; [^225^Ac]Ac) have a short path length and high linear energy transfer (12); this may be more suitable for overcoming radiation resistance in heavily pretreated disease (13), but for lesions with substantial heterogeneity of PSMA expression may theoretically lead to less durable responses due to untreated cells.

The novel, PSMA-targeted radiohybrid (rh) pharmaceutical rhPSMA-10.1 can be labelled with [^177^Lu]Lu or [^225^Ac]Ac. It has shown promising efficacy when labelled with [^177^Lu]Lu in preclinical models (14), and favourable radiation dosimetry and efficacy in preliminary studies of patients with mCRPC (15, 16). Ongoing phase 1/2 clinical trials are investigating the safety and efficacy of [^177^Lu]Lu-rhPSMA-10.1 in mCRPC (NCT05413850), in biochemical recurrence of prostate cancer (NCT06105918), and in high-risk, localized prostate cancer (NCT06066437 [the Nautilus trial]).

Preclinical investigation of [^225^Ac]Ac-labelled PSMA-targeted RPT has been conducted (17), and early clinical data have demonstrated biochemical and radiological responses to [^225^Ac]Ac-labelled PSMA-targeted RPT (18–20). However, outcomes from high quality, prospective clinical studies are still awaited, and comparisons with beta particles may be necessary to determine which patients will benefit from which isotope.

Here, we present results from a series of preclinical assessments designed to explore the potential of [^225^Ac]Ac-rhPSMA-10.1 as a novel, alpha-particle-emitting, PSMA-targeted RPT for metastatic prostate cancer.

## 2 Methods

**Figure 1:**
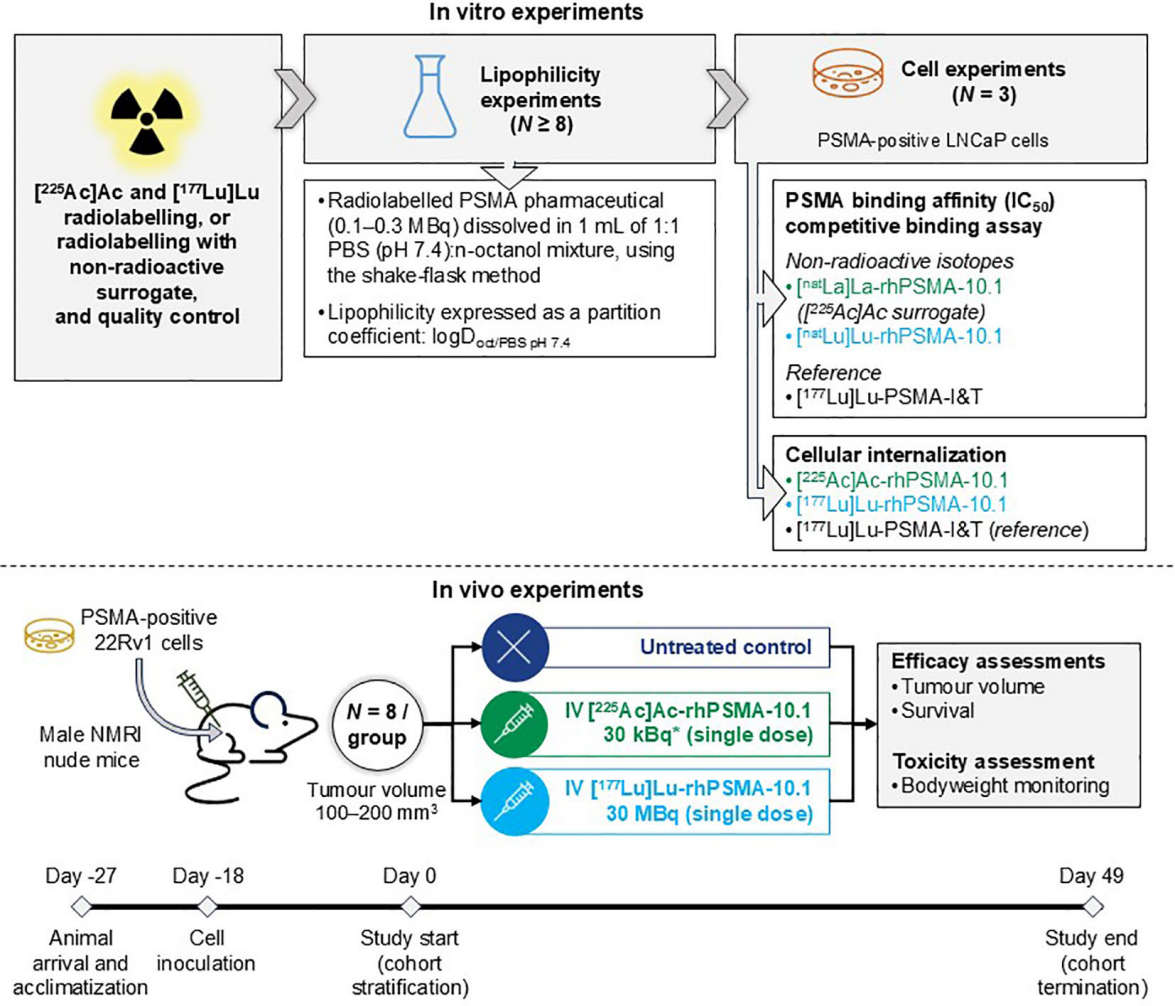
Study design. *1000-fold lower administered radioactivity for [^225^Ac]Ac-rhPSMA-10.1 determined based on data derived with [^225^Ac]Ac/[^177^Lu]Lu-PSMA-617 (Lee H. *Mol Imaging Radionucl Ther.* 2022;31:1–6). [^177^Lu]Lu, Lutetium-177; 22Rv1, human prostate adenocarcinoma cell line; [^225^Ac]Ac, Actinium-225; IC_50_, half-maximal inhibitory concentration; IV, intravenous; KBq, kilobecquerel; LNCaP, lymph node carcinoma of the prostate; MBq, megabecquerel; [^nat^La]La, natural Lanthanum; [^nat^Lu]Lu, natural Lutetium; NMRI, Naval Medical Research Institute; oct, *n-*octanol; PBS, phosphate-buffered saline; PSMA, prostate-specific membrane antigen; rh, radiohybrid.

We first conducted a series of *in vitro* experiments to assess the properties of [^225^Ac]Ac-rhPSMA-10.1 and [^177^Lu]Lu-rhPSMA-10.1 radiopharmaceuticals in PSMA-expressing cell lines (LNCaP; lymph node carcinoma of the prostate). Next, we assessed the therapeutic responses to [^225^Ac]Ac-rhPSMA-10.1 and [^177^Lu]Lu-rhPSMA-10.1 in PSMA-expressing prostate cancer human xenograft mouse models derived from a human prostate adenocarcinoma cell line (22Rv1) (**Figure 1**).

Full details on the processes for radiolabelling rhPSMA-10.1 with [^225^Ac]Ac and [^177^Lu]Lu (conducted based on methodology previously published by Wurzer et al. (21)) are provided in the **Supplemental Materials**.

### 2.1 *In vitro* experiments

#### 2.1.1 Lipophilicity assessments

Lipophilicity of the rhPSMA-10.1 molecule radiolabelled with [^225^Ac]Ac and [^177^Lu]Lu (0.1–0.3 MBq of each radiopharmaceutical) was assessed by the shake-flask method as described previously (21). The lipophilicity experiment was repeated ≥ 8 times for each radiopharmaceutical. Lipophilicity was expressed as a partition coefficient between *n-*octanol and phosphate-buffered saline (log D_oct/PBSpH 7.4_).

#### 2.1.2 Cell experiments

PSMA binding affinity and cellular internalization experiments were conducted in the LNCaP cell line (ACC 256, ordered from DSMZ German Collection of Microorganisms and Cell Cultures GmBH, Braunschweig, Germany), an androgen-sensitive prostate cancer model that shows high levels of PSMA expression (22). Cell lines were maintained in complete Roswell Park Memorial Institute (RPMI) media (10% foetal bovine serum, L-Glutamine, Penicillin/Streptomycin) and were harvested 24 ± 2 hours prior to experiments, and seeded (1.5 × 10^5^ cells in 1 mL/well [PSMA binding affinity experiments] or 5 × 10^5^ cells in 1 mL/well [cellular internalization experiments]) in poly-*L*-lysine-coated 24-well plates (Greiner Bio-One, Austria).

#### 2.1.3 PSMA binding affinity

The rhPSMA-10.1 molecule was complexed with natural Lanthanum ([^nat^La]La), used as a non-radioactive [^225^Ac]Ac-surrogate per previous studies (23, 24), and a non-radioactive isotope of Lutetium ([^nat^Lu]Lu). The PSMA binding affinity of these non-radioactive equivalents was measured by determining the half-maximal inhibitory concentration (IC_50_) in a competitive binding assay, as described previously (25). [^177^Lu]Lu-PSMA-I&T was used as a reference radiopharmaceutical.

#### 2.1.4 Cellular internalization

Experiments were carried out based on previous methodology (25). Full details are provided in the **Supplemental Methods**.

### 2.2 *In vivo* experiments

All animal experimentation was carried out under a license (number 2016-15-0201-00920) approved by the National Animal Experiments Inspectorate under the Ministry of Environment and Food of Denmark.

*In vivo* analyses used the 22Rv1 cell line (Sigma Aldrich) as an androgen-independent model of human castration-resistant prostate adenocarcinoma cells with moderate and heterogenous PSMA expression (26). 22Rv1 cells were maintained in RPMI-1640 media (10% foetal calf serum, 1% penicillin/streptomycin) prior to inoculation.

Male Naval Medical Research Institute nude mice (supplied by Janvier labs, France) received a subcutaneous inoculation of 3 × 10^6^ 22Rv1 cells in 100 µL PBS/Matrigel at the flank at approximately 8 weeks of age. Mice with a tumour volume of 100–200 m^3^ were randomized into three treatment groups of 8 individuals: intravenous [^225^Ac]Ac-rhPSMA-10.1 30 KBq (single dose); intravenous [^177^Lu]Lu-rhPSMA-10.1 30 MBq (single dose); and untreated control. The 1000-fold lower dose for [^225^Ac]Ac-rhPSMA-10.1 was determined based on data derived with [^225^Ac]Ac/[^177^Lu]Lu-PSMA-617 (27).

Animals were randomized and treatment commenced on Day 0, and the study ended on Day 49. Efficacy was assessed by relative tumour growth and survival of mice versus untreated controls ≤ 49 days post-treatment initiation. Tumour size was measured 2–3 times per week with callipers, and tumour volume was calculated using the formula 0.52 (length × width^2^); relative tumour volume was calculated as fold change from Day 0 to Day 49. Animals with a tumour volume > 1500 mm or ≥ 10% of their total bodyweight, or with a bodyweight loss of > 20% from baseline were humanely euthanized.

Compound tolerability was assessed via bodyweight monitoring throughout the study. Relative bodyweight was calculated as percentage change in bodyweight from Day 0.

#### 2.2.1 Data analysis

Survival analysis (using Kaplan–Meier survival curves and log-rank Mantel–Cox survival analyses) was performed from Day 1 to study termination (*p* ≤ 0.05 was considered statistically significant). Tumour volume between treatments was compared using a two-way ANOVA and Tukey’s multiple comparisons test (data analysed until ≥ 3 mice remained per group).

## 3 Results

### 3.1 *In vitro* experiments

**Figure 2:**
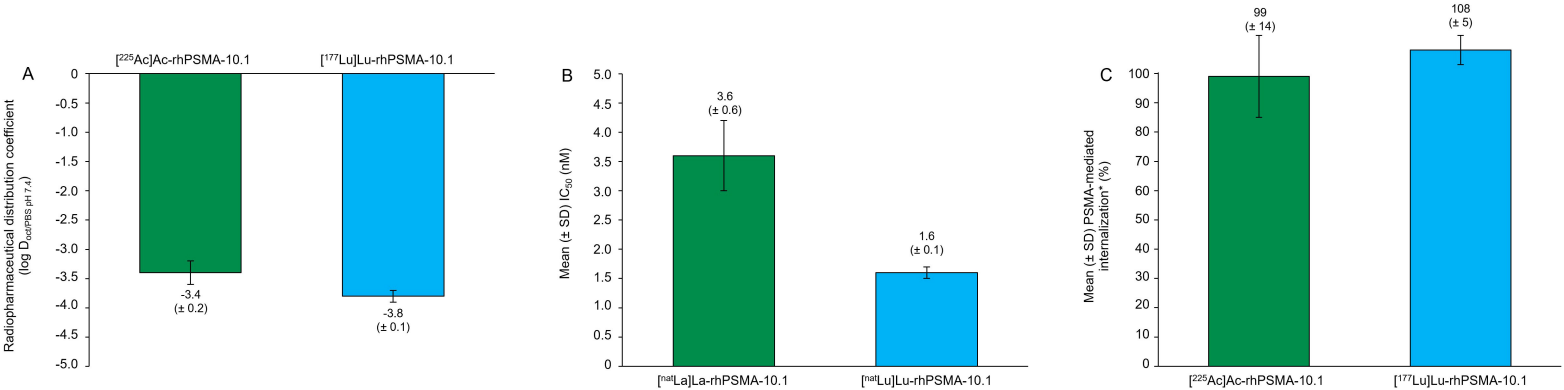
Results of *in vitro* experiments with [^225^Ac]Ac-rhPSMA-10.1 and [^177^Lu]Lu-rhPSMA-10.1 to determine **(A)** lipophilicity; **(B)** PSMA binding affinity (using non-radioactive surrogates); and **(C)** cellular internalization. PSMA binding affinity and cellular internalization were determined in the LNCaP human prostate cancer adenocarcinoma cell line (an androgen-sensitive model that shows high levels of PSMA expression). *Data are expressed as % internalization of the reference radiopharmaceutical ([^177^Lu]Lu-PSMA-I&T) and are corrected for non-specific binding (10 µmol phosphonomethyl pentandioic acid). [^177^Lu]Lu, Lutetium-177; [^225^Ac]Ac, Actinium-225; IC_50_, half-maximal inhibitory concentration; LNCaP, lymph node carcinoma of the prostate; [^nat^La]La, natural Lanthanum; [^nat^Lu]Lu, natural Lutetium; PSMA, prostate-specific membrane antigen; rh, radiohybrid; SD, standard deviation.

[^225^Ac]Ac-rhPSMA-10.1 and [^177^Lu]Lu-rhPSMA-10.1 had similar low lipophilicities (log D_oct/PBS pH 7.4_ = -3.4 ± 0.2 and log D_oct/PBS pH 7.4_ = -3.8 ± 0.1, respectively) (**Figure 2A**).

[^nat^La]La-rhPSMA-10.1 showed high PSMA binding affinity to PSMA-expressing LNCaP cells (IC_50_ = 3.6 ± 0.6 nM), with [^nat^Lu]Lu-rhPSMA-10.1 also showing a binding affinity in the low nM range (1.6 ± 0.1 nM) (**Figure 2B**).

The rate of cellular internalization into LNCaP cells at 1 hour, expressed as a percentage of the internalization of the reference radiopharmaceutical, was high for [^225^Ac]Ac-rhPSMA-10.1 (99 ± 14) and [^177^Lu]Lu-rhPSMA-10.1 (108 ± 5) (**Figure 2C**).

### 3.2 *In vivo* experiments

**Figure 3:**
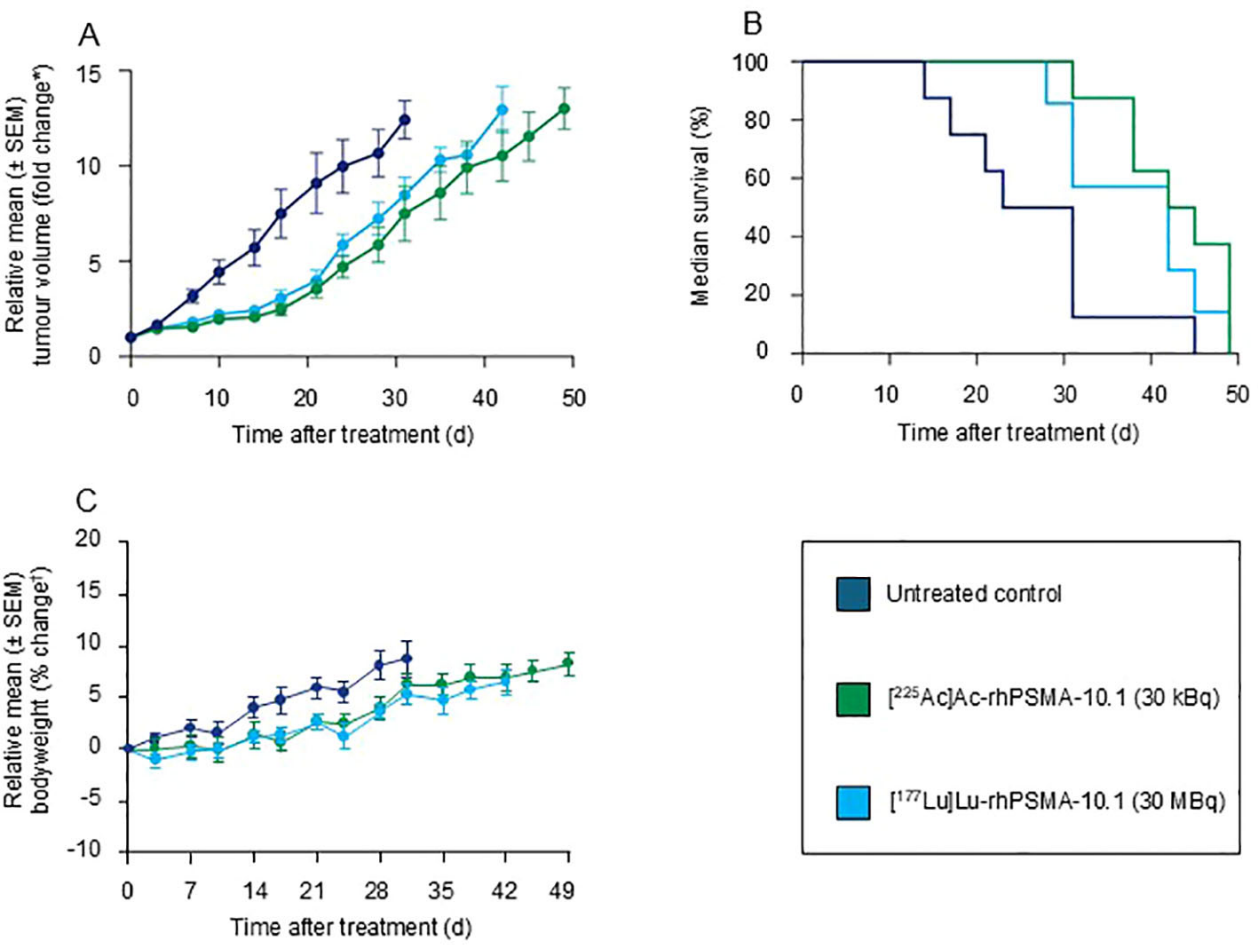
Therapeutic efficacy and tolerability of a single injection of [^225^Ac]Ac-rhPSMA-10.1 or [^177^Lu]Lu-rhPSMA-10.1 in PSMA-expressing 22Rv1 xenografts: **(A)** mean relative tumour volume; **(B)** median survival; **(C)** mean relative change in bodyweight across the study period. *N* = 8 per group and carry forward values until a minimum of 3 animals remained per group. Animals were sacrificed when the tumour volume endpoint was reached (Tumour volume > 1500 mm^3^). One mouse from the [^177^Lu]Lu-rhPSMA-10.1 group reached humane endpoint at Day 31, unrelated to tumour burden or treatment (*n* = 7 from Day 31), and was excluded from the survival analysis. *Fold change from Day 0 to Day 49. ^†^Percent change from Day 0 (study initiation). [^177^Lu]Lu, Lutetium-177; [^225^Ac]Ac, Actinium-225; 22Rv1, human prostate adenocarcinoma cell line; KBq, kilobecquerel; MBq, megabecquerel; PSMA, prostate-specific membrane antigen; rh, radiohybrid; SEM, standard error of the mean.

From Day 14 to Day 31, [^225^Ac]Ac-rhPSMA-10.1 and [^177^Lu]Lu-rhPSMA-10.1 both significantly reduced tumour growth compared with untreated controls (two-way ANOVA, Tukey’s multiple comparisons test: both *p* < 0.05). There was no significant difference in tumour growth suppression between [^225^Ac]Ac-rhPSMA-10.1 30 KBq and [^177^Lu]Lu-rhPSMA-10.1 30 MBq (**Figure 3A**).

[^225^Ac]Ac-rhPSMA-10.1 significantly prolonged median survival compared with untreated controls (43.5 days vs. 27.0 days, respectively; log-rank test, *p* = 0.006). Differences in median survival between [^177^Lu]Lu-rhPSMA-10.1 30 MBq (42.0 days) and [^225^Ac]Ac-rhPSMA-10.1 30 KBq (43.5 days), and [^177^Lu]Lu-rhPSMA-10.1 (42.0 days) and untreated control (27.0 days), were not statistically significant (**Figure 3B**).

[^225^Ac]Ac-rhPSMA-10.1 and [^177^Lu]Lu-rhPSMA-10.1 were both well tolerated, and no significant weight loss was observed with either treatment (**Figure 3C**).

## 4 Discussion

There is a need for novel treatments to prevent disease progression and improve the outcomes of men with metastatic prostate cancer (4). The novel [^177^Lu]Lu-labelled PSMA-targeted RPT rhPSMA-10.1 has been developed with the aim of optimized therapeutic properties (21, 28) and early data support a favourable therapeutic index in patients with mCRPC (15, 16). However, despite the proven safety and efficacy, and commercial availability of PSMA-targeted, [^177^Lu]Lu-labelled RPT in mCRPC, not all patients will exhibit favourable responses to treatment (4, 8) and it is estimated that at least one-third will not respond (8). Alpha-particle-emitting radiopharmaceuticals (such as those labelled with ^225^Ac) may thus be a feasible alternative for patients who progress on, do not respond, or develop resistance to [^177^Lu]Lu-labelled PSMA-targeted RPT (4, 10, 17, 19).

The different properties of beta- and alpha-particle-emitting radiation support the potential use of [^225^Ac]Ac-labelled PSMA-targeted RPT in a population of patients who do not benefit from and/or who progress on [^177^Lu]Lu-labelled PSMA-targeted RPT. [^177^Lu] has an estimated mean path length of 0.23 mm in soft tissue (29), which is sufficient to encompass the diameter of many cells. Therefore, theoretically, [^177^Lu] can induce DNA damage in non-target expressing cells via the ‘crossfire effect’, which may be useful for treating more heterogenous tumours (9). Additionally, early clinical data (17, 18) suggest less on-target normal tissue toxicity, for example caused by PSMA expression in the salivary glands. However, the low linear energy transfer of [^177^Lu] (approximately 0.34 keV/µm) (30) may also require higher radioactivity concentration in tumour lesions to effectively control disease (9). In contrast to [^177^Lu], the alpha particles generated by [^225^Ac] decay have shorter path lengths, ranging from approximately 47 to 85 µm in tissue (31) that may span < 5 cell diameters, and a higher linear energy transfer of approximately 80 keV/µm (32). These properties lead to complex DNA damage that is difficult for the cell to repair (9). This may better overcome radiation resistance mechanisms compared with beta-particle-emitting radiation, however, early clinical data (17, 18) suggest more profound normal tissue loss where the target of interest is expressed, for example, irreversible on-target damage to the salivary glands (17). There are also concerns about the biodistribution of dislocated [^225^Ac]Ac daughters to normal tissues, referred to as translocation, in [^225^Ac]Ac-labelled radiopharmaceuticals (33). Recent data indicate that translocation may be primarily driven by decays occurring during plasma circulation (34); if this is the case, a radiopharmaceutical with a shorter effective half-life in plasma could offer an opportunity to reduce absorbed doses delivered to normal tissues.

There is growing interest in the use of [^225^Ac]Ac-labelled, PSMA-targeted RPT in metastatic prostate cancer, including for compassionate use (35): several studies with [^225^Ac]Ac-PSMA-617 have shown therapeutic benefits in heavily pretreated patients with mCRPC (18–20, 35), including those who progressed on [^177^Lu]Lu-PSMA-617 (19), and a clinical study of [^225^Ac]Ac-PSMA-I&T in advanced mCRPC has demonstrated comparable antitumour activity to studies of [^225^Ac]Ac-PSMA-617 (36). However, despite early promising data that have emerged from compassionate use, particularly in South Africa and India, it remains unclear whether [^225^Ac]Ac labelling represents an improvement in the benefit-risk profile compared with the currently available commercial option ([^177^Lu]Lu vipivotide tetraxetan) (37). Notably, there are reports of irreversible salivary gland toxicity leading to high levels of treatment discontinuation (38), and, in some cases, renal failure (39) and follow up clinical studies with [^225^Ac]Ac are required to fully assess the long-term safety profile. To date, much data has been collected retrospectively (outside of formal clinical trials) and many of the patients exposed in these experiences have not received the full spectrum of currently available prostate cancer treatments in the United States, which has made it difficult to assess whether the early results are replicable in patients from the United States and Europe. However, this situation is changing following recent publications reporting data from Phase 1 and 2 clinical trials investigating [^225^Ac]Ac-PSMA-RPT, with additional trials currently ongoing.

Early phase clinical trials investigating several small molecule [^225^Ac]Ac-PSMA-RPTs have demonstrated a tolerable safety profile and have reported efficacy in men with mCRPC, as assessed by a decline in PSA. The AcTION Phase 1 dose-escalation trial (NCT04597411), investigating [^225^Ac]Ac-PSMA-617 in patients with mCRPC recently reported an acceptable safety profile, with no dose-limiting toxicities (DLTs), and a dry-mouth the most common treatment-related adverse event (TRAEs), no patients experienced Grade 4 or 5 TRAEs. Moreover, a ≥ 50% decline in PSA (PSA50) was achieved in 53% of patients who had undergone prior [^177^Lu]Lu-PSMA-RPT (40). Similarly, the Phase 1 PAnTHa trial (NCT06217822) investigating [^225^Ac]Ac-PSMA-Trillium in patients with mCRPC reported that [^225^Ac]Ac-PSMA-Trillium was well tolerated with no DLTs, and a dry-mouth was the most common treatment-emergent adverse event (TEAE). Overall, 16% of patients had serious TEAE. Notably, a PSA50 was reported for 83% of patients who underwent the recommended dose for expansion (41). Preliminary results from the Phase 2 TATCIST trial (NCT05219500) investigating the efficacy and safety of FPI-2265 ([^225^Ac]Ac-PSMA-I&T), in patients with mCRPC also showed a tolerable safety profile, a dry-mouth was the most common TRAE, and no Grade 4 or 5 TRAEs observed. A PSA50 was achieved in 50% of the 20 patients included in the efficacy analysis; most patients had heavily pre-treated disease, and eight had undergone prior [^177^Lu]Lu-PSMA-RPT (42). Although these studies are not directly comparable due to differing patient populations and treatment regimens. In addition, early phase trials have also investigated the use of [^225^Ac]Ac-labelled PSMA-targeting monoclonal antibodies. For example, the Phase 1 dose-escalation study (NCT03276572) of the PSMA-targeting monoclonal antibody [^225^Ac]Ac-J591 reported a tolerable safety profile and a confirmed PSA50 response was reported in 34% of patients (43). A further Phase 1/2 trial assessing [^225^Ac]Ac-J591 has recently reached completion (NCT04506567).

Large-scale, multicentre, prospective, randomized studies are required before any firm conclusions regarding the safety and efficacy of [^225^Ac]Ac-PSMA-RPT can be made. However, given the promising early safety and efficacy results from several Phase 1/2 trials involving [^225^Ac]Ac-PSMA-RPT, and considering the optimized properties of the rhPSMA-10.1 molecule, it is logical to assess its performance when labelled with [^225^Ac]Ac.

In this first study with an [^225^Ac]Ac-labelled, rh, PSMA-targeted RPT ([^225^Ac]Ac-rhPSMA-10.1), we established the *in vitro* properties of [^225^Ac]Ac-rhPSMA-10.1 in the LNCaP cell line compared with [^177^Lu]Lu-rhPSMA-10.1 and, subsequently, demonstrated efficacy of both radiopharmaceuticals in 22Rv1 xenografts. We selected the 22Rv1 xenograft model over the LNCaP model for *in vivo* analysis of [^225^Ac]Ac-rhPSMA-10.1 as we believed this model best represented the heterogeneous levels of PSMA expression seen in humans and represented a more stringent model for testing the therapeutic efficacy of PSMA-targeted radiopharmaceuticals (14, 26).

We showed PSMA binding affinity, lipophilicity, and cellular internalization values for [^225^Ac]Ac-rhPSMA-10.1 to be similar to [^177^Lu]Lu-rhPSMA-10.1, which is consistent with previous data for [^177^Lu]Lu-rhPSMA-10.1 (21). Based on the similarities between both radiopharmaceuticals observed in our *in vitro* analysis, we expected [^225^Ac]Ac-rhPSMA-10.1 and [^177^Lu]Lu-rhPSMA-10.1 to perform similarly when tested *in vivo*, albeit at radioactivity levels adjusted for the different biological effects of beta versus alpha particles. Indeed, both [^177^Lu]Lu-rhPSMA-10.1 and [^225^Ac]Ac-rhPSMA-10.1 reduced tumour growth and improved survival versus controls in prostate cancer 22Rv1 xenografts, and both were well tolerated. Importantly, we found that [^225^Ac]Ac-rhPSMA-10.1 had the same antitumour effects as [^177^Lu]Lu-rhPSMA-10.1 when delivered at a 1000-fold lower injected radioactivity in our preclinical model, reflecting the properties of alpha-emitting radiation. Our findings were consistent with a previous preclinical study of [^177^Lu]Lu-rhPSMA-10.1, which demonstrated therapeutic efficacy in 22Rv1 and LNCaP prostate cancer xenografts (14). Given the similar therapeutic effects of [^177^Lu]Lu-rhPSMA-10.1 and [^225^Ac]Ac-rhPSMA-10.1 in 22Rv1 xenografts observed in the current preclinical work, and the consistency of our [^177^Lu]Lu-rhPSMA-10.1-related findings with previous results (14), we would expect [^225^Ac]Ac-rhPSMA-10.1 to perform similarly to [^177^Lu]Lu-rhPSMA-10.1 in LNCaP xenografts.

As discussed, the efficacy between [^177^Lu]Lu-rhPSMA-10.1 (30 MBq) and [^225^Ac]Ac rhPSMA-10.1 (30 kBq) was broadly comparable in our xenograft model. This may be in part due to the relatively modest and heterogenous PSMA expression of the 22Rv1 model which may limit the ability to fully resolve potential advantages associated with alpha-particle therapy, particularly given the short path length and reduced cross-fire effect of alpha-emissions compared with beta. Clinically, alpha-particle-emitting radiation may be advantageous when used to overcome resistance following previous beta-particle-emitting therapies; however, this is not tested within our model. Furthermore, an *in vitro* study demonstrated that the difference in the efficacy per administered dose is much higher between alpha- and beta-emitting particles in microscopic compared with macroscopic disease, with alpha-emitting particles 3000–4000 times more potent than beta-emitting particles in microscopic disease (27). These data suggest that in a clinical setting alpha particles may have the potential to be more efficacious than beta particles in specific settings, including the treatment of occult metastases. Indeed, a Phase 1 trial investigating [^225^Ac]Ac-rhPSMA-10.1 in patients who have progressed after previous treatments, particularly after [^177^Lu]Lu-PSMA-RPT started enrolment in 2026 (NCT07414940).

There are some limitations to our work. Our findings suggested that the exclusion of one mouse from the [^177^Lu]Lu-rhPSMA-10.1 group that reached humane endpoint at Day 31, unrelated to tumour burden or treatment, could have impacted the statistical power of the survival analysis, given that the robust prolongation of median survival compared with control (42.0 days vs. 27.0 days, respectively) was not statistically significant. Indeed, [^177^Lu]Lu-rhPSMA-10.1 significantly prolonged median survival versus control in both 22Rv1 xenografts (with moderate and heterogeneous levels of PSMA expression) and LNCaP xenografts (with high levels of PSMA expression) in a previous study. Suppression of tumour growth by [^177^Lu]Lu-rhPSMA-10.1 was lower and less sustained in 22Rv1 than in LNCaP xenograft–bearing mice, likely because of the lower levels of PSMA expression (14). Whilst a limitation of this study is that we did not evaluate [^225^Ac]Ac-rhPSMA-10.1 efficacy in LNCaP xenografts, based on earlier work we believe 22Rv1 xenografts provided a rigorous model (14). However, the extent to which these findings are generalizable to other prostate cancer xenograft models with differing levels of PSMA expression would need to be confirmed. Similarly, we evaluated the efficacy of both [^177^Lu]Lu-rhPSMA-10.1 and [^225^Ac]Ac-rhPSMA-10.1 over a period of 49 days after a single dose. Future studies incorporating regimens with multiple doses may aid our understanding of the cumulative toxicity of these radiopharmaceuticals. Dosimetry and biodistribution analyses were beyond the scope of this study; however, these analyses will be required in the future to support the interpretation of [^225^Ac]Ac-rhPSMA-10.1 efficacy and safety data. Finally, a weakness of subcutaneous xenograft RPT mouse models is that tumours do not fully represent prostate cancer in humans, given the flank localization, the size differential versus humans, the density of PSMA expression (which is likely an order of magnitude higher compared with human cancer), and the fixed physical properties of the isotope. Therefore, caution should be applied when extrapolating results from mouse models to humans. Nevertheless, the promising results obtained in our preclinical evaluation support future clinical investigation of [^225^Ac]Ac-rhPSMA-10.1 as a novel, alpha-particle-emitting RPT for patients with metastatic prostate cancer.

Overall, results from our preclinical analyses demonstrated that [^225^Ac] labelling of rhPSMA-10.1 is feasible, yielding similar *in vitro* properties to the [^177^Lu]Lu-radiolabelled equivalent. [^225^Ac]Ac-rhPSMA-10.1 also delivered similar efficacy in prostate cancer xenografts, with lower administered radioactivity than [^177^Lu]Lu-rhPSMA-10.1. Our preclinical findings support the investigation of [^225^Ac]Ac-rhPSMA-10.1 as a novel, alpha-particle-emitting RPT in clinical trials of men with metastatic prostate cancer.

## Data Availability

The raw data supporting the conclusions of this article will be made available by the authors, without undue reservation.
