# Supplementary material for: Actinium-225-rhPSMA-10.1 as a novel, alpha-particle-emitting therapy for prostate cancer: results of a preclinical evaluation
Source: Front Oncol. 2026 Jul 16;16:1868487. doi: 10.3389/fonc.2026.1868487 (PMC13422538; doi:10.3389/fonc.2026.1868487)
Supplement: Supplementary file 1 [file DataSheet1.docx]

# Supplementary Material

## 1 Radiolabeling Methodology

*1.1 Actinium-225 ([^225^Ac]Ac) Radiolabeling*

For in vitro analyses, [^225^Ac] was obtained as a solid film (Joint Research Centre, Karlsruhe, Germany) that was dissolved in 50 µL of 0.1 M hydrochloric acid (HCl) (Suprapur, Merck) and incubated at room temperature for ≥ 12 hours to ensure complete dissolution. [^225^Ac] delivered in 0.04 M HCl (Isotope Technologies Munich) was also used. [^225^Ac]-labelling reactions were performed either in sealed or crimped 0.5–2.0 mL glass vials (Biotage), using a Biotage Initiator^+^ micro wave reactor, or in a 1.5 mL Eppendorf Safe‑lock tube using a heating block, using an acetate buffer prepared with anhydrous > 99% sodium acetate (Sigma Aldrich), glacial high-performance liquid chromatography (HPLC)-grade acetic acid (Thermo Fisher Scientific), and aqueous HCl (Suprapur, Merck), in a total radiolabeling volume of approximately 0.1 mL/MBq using 10 nmol of rhPSMA-10.1 precursor per MBq of [^225^Ac].

For in vivo analyses, peptide from stock (Almac) was added to reach the desired specific labelling activity, and the reaction mixture was stirred for 30 minutes at 80 °C. The radionuclide incorporation was determined by radio thin-layer chromatography (TLC). For radio-TLC, the plate was cut in half, and each half was immediately measured twice in a γ‑counter. The [^225^Ac]Ac activity counts were estimated based on the Francium-221 and Bismuth-213 peak in the spectrum. The anticipated volume per injection of the final product was 0.15 mL (30 KBq activity) and the formulation was prepared to an appropriate activity concentration for this target.

Radiochemical purity in the final product at end of the in vivo study was measured by radio-TLC. The plate was cut in half, and each half was measured in a γ‑counter. The [^225^Ac] counts were measured after secular equilibrium was reached. A sample was taken for radio-HPLC analysis at the end-of-synthesis. Fractions from the radio-HPLC analysis were collected and measured on the well counter. A sample from the final product was re-analyzed by radio-HPLC after the final animal had been injected. Fractions of 0.5 mL from the radio-HPLC analysis were collected and measured on the well counter 24 hours after collection when secular equilibrium was reached. Analyzed fractions were used to determine the radiochemical purity of the final product at the end-of-synthesis and after the final animal had been injected.

*1.2 Lutetium-177 ([^177^Lu]) Radiolabeling*

For in vitro analyses, [^177^Lu] precursor (1.0 nmol, 10 μL, 0.1 mM in dimethyl sulfoxide) was added to 10 μL of 1.0 M aqueous acetate buffer (pH 5.5) prepared with anhydrous > 99% sodium acetate (Sigma Aldrich), glacial HPLC-grade acetic acid (Thermo Fisher Scientific) and aqueous HCl (Suprapur, Merck).

Subsequently, 30-40 MBq of Lutetium chloride ([^177^Lu]LuCl_3_; specific activity > 3000 GBq/mg, 740 MBq/mL, 0.04 M HCl) (ITM, Garching, Germany) was added and the mixture filled up to 100 μL with water. The reaction mixture was heated for 20 minutes at 90 °C and the radiochemical purity was determined using radio-HPLC and radio-TLC without any further formulation performed, as described previously [1].

The optimized protocol for [^177^Lu]Lu-labelling of rhPSMA-10.1 for the in vivo analysis was based on a previous study [1]. The compound was labelled at a specific activity of 60 MBq/nmol. [^177^Lu] was added, the activity was measured, and ascorbate buffer added (activity in MBq/1.8 = volume labelling buffer).

The peptide was added to reach the desired specific activity using a 1 mg/mL solution. pH in the reaction was measured by pH indicator paper (up to pH 4), and the reaction mixture was stirred for 15 minutes at 80 °C. The product was formulated to a concentration of 200 MBq/mL (30 MBq/150 μL) in ascorbate buffer containing diethylenetriaminepentaacetic acid in TraceSelect water (pH 5.0) (Minerva Imaging).

Radio-TLC and radio‑HPLC were performed after the last in vivo injection was completed to verify the quality of the radiolabeled product injected (radionuclide incorporation > 98%).

## 2 Cellular Internalization Methodology

After harvesting and removal of the culture medium, plated LNCaP cells were washed once with 500 μL Roswell Park Memorial Institute (RPMI) media (5% bovine serum albumin [BSA]) and left to equilibrate for ≥ 15 minutes at 37 °C in 200 μL RPMI media (5% BSA). During incubation, [^225^Ac]Ac‑labeled rhPSMA-10.1 was purified by solid‑phase extraction in a cartridge-based purification step to remove the daughter nuclides that were in secular equilibrium with [^225^Ac]Ac-rhPSMA-10.1 at the time purification started. Each well was treated with 25 μL of either RPMI (5% BSA, control) or 25 μL of a 100 μM 2‑(phosphonomethyl)-pentandioic acid (2-PMPA; Tocris Bioscience, United Kingdom) solution in phosphate buffered saline (PBS) for blockade. Next, 25 μL of the radioactive-labelled PSMA inhibitor ligand (50 nM in PBS) was added, and the cells were incubated at 37 °C for 60 minutes. The experiment was terminated by placing the 24-well plate on ice for 3 minutes and removal of the medium. Each well was carefully washed with 250 μL of ice-cold PBS. Both fractions from the first steps representing the amount of free radioligand were combined. Removal of surface-bound activity was accomplished by incubation of the cells with 250 μL of ice-cold PMPA (10 μM in PBS) solution for 15 minutes.

The internalized activity was determined by incubation of the cells in 250 μL of 1 M aqueous sodium hydroxide (NaOH) for at least 10 minutes. The obtained fractions were combined with those of the subsequent washing step with 250 μL of 1 M aqueous NaOH. Each experiment (control and blockade) was performed in triplicate. Free, surface-bound, and internalized activity was quantified in a γ-counter. All internalization studies were complemented by external reference studies using the reference compound (50 nM [^177^Lu]Lu-PSMA-I&T), which were performed analogously. Data were corrected for non-specific binding by subtracting the “amount of internalized PSMA-ligand in the presence of PMPA = blockade” from the “amount of internalized PSMA-ligand = total uncorrected internalization”. Finally, the internalization was normalized to the specific internalization observed for the reference compound.

## Reference

**1.** Wurzer A, Kunert JP, Fischer S, et al. Synthesis and preclinical evaluation of ^177^Lu-labeled radiohybrid PSMA ligands (rhPSMAs) for endoradiotherapy of prostate cancer. *J Nucl Med.* 2022;63:1489–1495.
